# Liver × receptor ligands disrupt breast cancer cell proliferation through an E2F-mediated mechanism

**DOI:** 10.1186/bcr3443

**Published:** 2013-06-20

**Authors:** Trang Nguyen-Vu, Lise-Lotte Vedin, Ka Liu, Philip Jonsson, Jean Z Lin, Nicholes R Candelaria, Lindsay P Candelaria, Sridevi Addanki, Cecilia Williams, Jan-Åke Gustafsson, Knut R Steffensen, Chin-Yo Lin

**Affiliations:** 1 Center for Nuclear Receptors and Cell Signaling, Department of Biology and Biochemistry, University of Houston, 3605 Cullen Boulevard, Houston, TX, 77204-5506, USA; 2 Department of Biosciences and Nutrition at NOVUM, Karolinska Institute, Hälsovägen 9, S-14183 Huddinge, Sweden; 3 Center for Diabetes Research, The Methodist Hospital Research Institute, The Methodist Hospital, 6565 Fannin Street, Houston, TX, 77030, USA

**Keywords:** Liver × receptor, nuclear receptor, ligand, microarray, E2F, breast cancer

## Abstract

**Introduction:**

Liver × receptors (LXRs) are members of the nuclear receptor family of ligand-dependent transcription factors and have established functions as regulators of cholesterol, glucose, and fatty acid metabolism and inflammatory responses. Published reports of anti-proliferative effects of synthetic LXR ligands on breast, prostate, ovarian, lung, skin, and colorectal cancer cells suggest that LXRs are potential targets in cancer prevention and treatment.

**Methods:**

To further determine the effects of LXR ligands and identify their potential mechanisms of action in breast cancer cells, we carried out microarray analysis of gene expression in four breast cancer cell lines following treatments with the synthetic LXR ligand GW3965. Differentially expressed genes were further subjected to gene ontology and pathway analyses, and their expression profiles and associations with disease parameters and outcomes were examined in clinical samples. Response of E2F target genes were validated by real-time PCR, and the posited role of E2F2 in breast cancer cell proliferation was tested by RNA interference experiments.

**Results:**

We observed cell line-specific transcriptional responses as well as a set of common responsive genes. In the common responsive gene set, upregulated genes tend to function in the known metabolic effects of LXR ligands and LXRs whereas the downregulated genes mostly include those which function in cell cycle regulation, DNA replication, and other cell proliferation-related processes. Transcription factor binding site analysis of the downregulated genes revealed an enrichment of E2F binding site sequence motifs. Correspondingly, E2F2 transcript levels are downregulated following LXR ligand treatment. Knockdown of E2F2 expression, similar to LXR ligand treatment, resulted in a significant disruption of estrogen receptor positive breast cancer cell proliferation. Ligand treatment also decreased E2F2 binding to cis-regulatory regions of target genes. Hierarchical clustering of breast cancer patients based on the expression profiles of the commonly downregulated LXR ligand-responsive genes showed a strong association of these genes with patient survival.

**Conclusions:**

Taken together, these results indicate that LXR ligands target gene networks, including those regulated by E2F family members, are critical for tumor biology and disease progression and merit further consideration as potential agents in the prevention and treatment of breast cancers.

## Introduction

Advances in breast cancer therapy are facilitated by molecular characterizations of tumors and tumor subtypes. For example, breast tumors that express estrogen receptor (ER-positive, ER+) and progesterone receptor (PR-positive, PR+) and are dependent on the female sex hormone estrogen for growth and proliferation are treated by drugs that target ER either directly (tamoxifen, raloxifene, fulvestrant) or indirectly (letrozole, anastrozole, exemestane) by disrupting estrogen production [1,2]. Tumors that overexpress human epidermal growth factor receptor 2 (HER2/ErbB-2/neu+) on cell surfaces are targeted by monoclonal antibodies (trastuzumab) or tyrosine kinase inhibitors (lapatinib), which block receptor activation and tumor cell proliferation [3]. Some tumors, however, are refractory to these targeted therapies or develop resistance over time. Blocking estrogen production and functions also imparts menopausal symptoms and increases corresponding health risks in premenopausal women. Moreover, a significant number of patients have triple negative (ER-, PR-, and HER2-) breast cancers and require alternative targeted chemopreventive and therapeutic strategies [4]. Improvements of current breast cancer therapeutics and development of new ones necessitate the discovery and characterization of novel target mechanisms and targeting agents.

Both ER and PR belong to the nuclear receptor (NR) superfamily of ligand-dependent transcription factors, which function in normal development and physiology and in a number of human diseases [5]. NRs are highly druggable targets of synthetic and natural ligands, and accumulating insights on NR structures and functions and advances in medicinal chemistry have provided receptor-selective, full, partial and inverse agonists and antagonists, as well as compounds that activate only a subset of the NR functions or in a tissue-specific manner. Discovery and characterization of other NRs that may also play important roles in breast cancer biology are, therefore, likely to yield other promising target mechanisms and agents.

Liver × receptors (LXRs) are NRs that are activated by oxysterols, synthetic ligands, and dietary phytosterols and have been well characterized as regulators of cholesterol, glucose, and fatty acid metabolism and inflammatory responses [6-12]. At the molecular level, two receptor subtypes, LXRα and LXRβ, function in heterodimers with 9-cis retinoic acid receptors (RXRs), and their activities as transcriptional regulators are modulated by ligand binding and post-translational modifications mediated by cell signaling pathways [13]. A number of LXR ligands have been developed for the treatment of atherosclerosis, diabetes, Alzheimer's disease, and inflammation. Published reports of anti-proliferative effects of LXR ligands on breast, prostate, ovarian, lung, skin, and colorectal cancer cells suggest that LXRs are potential targets in cancer prevention and treatment [14-17]. Observations of increased proliferation markers in colon tissues and pre-neoplastic lesions in the gallbladder of LXRβ knock-out animals further suggest a role for LXRs and their ligands in cancer initiation and progression [18,19]. We have previously shown that synthetic LXR ligands can block the proliferation of both ER+ and ER- breast cancer cells through downregulation of some cell cycle and growth-associated genes [20]. To additionally determine the effects of LXR ligands on breast cancer cells and to identify their potential mechanisms of action, we carried out microarray analysis of gene expression following treatment with the synthetic LXR ligand GW3965 in multiple breast cancer cell lines. Here, we report our findings regarding the effects of LXR activation on breast cancer transcriptomes, the potential role of E2F2 in mediating the anti-proliferative effects in ER+ breast cancer cells, and association of ligand-responsive gene networks with disease outcomes in breast cancer patients.

## Materials and methods

### Cell culture and treatment

MCF-7 ER+ breast cancer cells were cultured in DMEM (Invitrogen, Carlsbad, CA, USA) supplemented with 10% FBS (Saveen Werner, Limhamn, Sweden or Hyclone, Logan, UT, USA). ER+ T-47D cells were cultured in DMEM:F12 (Invitrogen) supplemented with 5% FBS. ER- SK-BR-3 and MDA-MB-231 cells were grown in Roswell Park Memorial Institute (RPMI) 1640 medium (Invitrogen) supplemented with 10% FBS. For microarray analysis, cells were treated with 10 μM of the synthetic LXR ligand GW3965 for 48 hours before harvest and RNA isolation with EZNA Total RNA Kit I (Omega Bio-Tek, Norcross, GA, USA) according to the manufacturer's protocol. Current institutional and governmental safety guidelines were followed in the performance of these experiments, and no additional ethical approval was required from institutional review boards.

### Microarray and data analysis

We amplified 250 ng of RNA, which was converted to cRNA using the Illumina TotalPrep-96 RNA Amplification kit (Ambion, Carlsbad, CA, USA): 750 ng of cRNA was used for hybridization onto the Illumina Whole-Genome Gene Expression Direct Hybridization microarray (Illumina, San Diego, CA, USA), and 25,559 probes from the microarray were included for analysis. Probes for multiple genes were eliminated. The R software running the lumi and limma packages were used to determine differentially expressed genes in ligand-treated cells. Normalized intensity values were log-2 transformed. To correct for false discovery, we implemented the Benjamini-Hochberg correction [21]. An additional filter for differentially expressed genes was set at 1.5-fold change in expression in either direction. Array data have been deposited in the NCBI Gene Expression Omnibus database [GSE34987].

### Data mining

Bioinformatic analyses of enriched gene sets were made in Pathway Studio (Ariadne Genomics, Rockville, MD, USA). Fisher's exact test was applied to determine significantly enriched pathways. The gene sets for analysis of transcription factor (TF) target enrichment, TFT version 3.0, were downloaded from the Broad Institute [22], whereas the gene ontology (GO) categories used were provided within the software. TFT 3.0 contains sets of genes that share a common predicted TF binding site as defined in TRANSFAC version 7.4. The enrichment of E2F motifs was compared to a random sampling of promoters for all transcription factors in the MSigDb database. The promoter region was defined to be 2kb up- or downstream of the transcription start site.

### Quantitative PCR

For time-course experiments, cells were plated in 6-well plates and treated with 5 μM GW3965 for 6 to 72 hours before harvest and RNA isolation. cDNA synthesis was performed using SuperScript II reverse transcriptase (Invitrogen). Quantitative PCR (qPCR) was carried out using Fast SYBR Green Master Mix (Applied Biosystems, Carlsbad, CA, USA) in the 7500 fast real-time PCR system (Applied Biosystems). Primers (Additional file 1) for specific genes were designed using Primer3 software. Relative transcript levels were calculated using the ΔΔct (cycle threshold) method with 36B4 as the reference gene. Statistical significance was determined by Student's *t*-test.

### RNA interference

MCF-7, T-47D, and MDA-MB-231 cells were seeded in 6-well plates (9.6 cm^2^, area per well) in appropriate media mentioned above. After 24 hours, cells were washed with PBS and transfected with either 10 nM E2F2 siRNA (Dharmacon, Lafayette, CO, USA) or 10 nM non-targeting control (Dharmacon), using DharmaFECT I transfection reagent (Dharmacon). Cells were counted using the trypan blue staining method and the Countess automated cell counter (Invitrogen). To validate gene knockdown, RNA isolation, cDNA synthesis and qPCR were performed in the same way as described above.

### Western blot analysis

After 48 hours of E2F2 siRNA or non-targeting control treatment, cells were lysed with RIPA lysis buffer. Protein concentrations were then determined using Qubit^® ^Protein Assay Kit and fluorometer (Invitrogen, New York, NY, USA). Total protein (100 μg from each sample) was loaded onto a 10% polyacrylamide gel. After electrophoresis, proteins separated by SDS-PAGE were transferred to a polyvinylidene fluoride (PVDF) membrane (Millipore, Billerica, MA, USA). The membrane was blocked with 5% nonfat milk in Tris-buffered saline and Tween 20 (TBST) and then incubated with antibodies against E2F2 (Santa Cruz Biotechnology, Inc., Santa Cruz, CA, USA) or β-actin (catalog number: A2228, Sigma, St. Louis, MO, USA) in TBST overnight. The membrane was then reprobed with appropriate secondary antibodies conjugated with horseradish peroxidase for 1 hour. Blots were processed using an ECL kit (Thermo Fisher Scientific, Rockford, IL, USA) and exposed to film.

### Chromatin immunoprecipitation

Chromatin immunoprecipitation (ChIP) assays were performed as described previously [23,24]. Immunoprecipitations were carried out with E2F2 antibody (Santa Cruz Biotechnology, Santa Cruz, CA, USA) or corresponding pre-immune IgG (Santa Cruz Biotechnology). Binding of E2F2 to response elements was measured by qPCR using specific primers (Additional file 1).

### Clustering of clinical samples and survival analysis

Clinical and microarray gene expression data from a previously published breast cancer study cohort from Uppsala, Sweden, were used to determine the correlation of the expression profiles of the 83 commonly responsive genes with clinical parameters and disease outcomes [25]. Dendrograms and heat maps were generated using the Eisen Cluster and Treeview software. Survival data were analyzed using Kaplan-Meier plot functions (log-rank test) of the SigmaPlot software. These analyses utilized previously published and publicly available clinical and experimental data from patient samples and therefore no consent or institutional review was required.

## Results

### Global effects of LXR ligand treatment on gene expression

Previous studies of LXR ligands and LXRs in cancer cells have focused on their effects on cell cycle regulation and cell proliferation [14-17,20]. It is unclear, however, which LXR-regulated genes are involved in the observed disruption of cancer cell proliferation. To determine their transcriptomic effects on breast cancer cell gene expression and to identify potential target mechanisms of LXR ligands, we performed microarray analysis of gene expression in MCF-7, T-47D, SK-BR-3, or MDA-MB-231 cells, four well-characterized cell line models of ER+ and ER- breast cancers, which express both LXRα and LXRβ (Additional file 2) and have previously been shown to be sensitive to LXR ligand treatment [20], following either control treatments with dimethyl sulfoxide (DMSO) vehicle or GW3965 LXR ligand.

**Figure 1:**
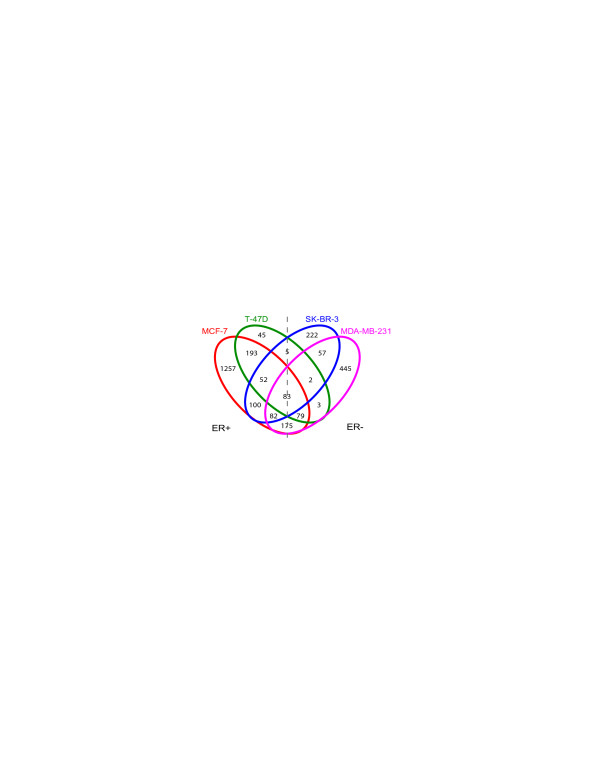
**Venn diagram of liver × receptor ligand-responsive genes across four different breast cancer cell lines**. Responsive genes were defined as those that were statistically significant (false-discovery rate-corrected *P *< 0.05) and at least 1.5-fold change in treated cells compared to vehicle-treated controls. ER+, estrogen receptor-positive; ER-, estrogen receptor-negative.

Using a false discovery rate-corrected *P*-value cutoff of <0.05 and at least a 1.5-fold increase or decrease in expression, a total of 2,800 genes showed differential expression in response to ligand treatments in at least one cell line as compared to vehicle-treated controls. The results are summarized in Figure 1. ER+ MCF-7 cells exhibited the most robust response with 2,021 differentially expressed genes. Only 462 genes were responsive to ligand treatment in ER+ T-47D cells, indicating significant genomic differences in transcriptional programming between cell lines in spite of their common ER status. Interestingly, ER (ERα) transcript levels decreased in response to LXR ligand treatment in both ER+ cell lines. This is consistent with previous observations of decreased ERα transcript and protein levels in ligand-treated MCF-7 cells and suggests a potential mechanism for modulating ER activity and cell proliferation in ER+ breast cancer cells by LXR ligands [20]. To further determine the impact of the downregulation of ER expression on the changes in gene expression following ligand treatment, we cross-referenced the 193 common ligand-responsive genes in the two ER+ cells lines and compared them to a list of 938 estrogen-responsive genes identified in previously published studies [26-29]. Of the 193 common LXR ligand-reponsive genes in the two ER+ cell lines, 25 are likely downstream targets of ER. In ER- breast cancer cells, 603 genes were responsive to ligand treatment in SK-BR-3 cells, and 926 genes were responsive in the triple-negative MDA-MB-231 cells. Of all the ligand-responsive genes, 83 (23 upregulated and 60 downregulated) were responsive in all four breast cancer cell lines, including known LXR target genes such as ABCA1 and ABCG1 [30,31], and they represent candidate mechanisms for the anti-proliferative effects of LXR ligands in these cell lines.

### Ligand treatment downregulates genes involved in cell growth and proliferation

Out of the 2,800 LXR ligand-responsive genes identified in our microarray study, 83 showed consistent responses across all four breast cancer cell lines examined (Additional file 3). We posited that these conserved responsive genes are likely involved in core LXR functions, which may include molecular and cellular processes related to known metabolic functions of LXRs and their ligands and also those involved in cell growth and proliferation, which are the focus of this study. To test this hypothesis, we carried out GO analysis of functional categories that are enriched in this commonly responsive gene set, using the Pathway Studio software package.

Since genes involved in the same pathways and processes are likely co-regulated, we first divided the set of 83 genes into those that were upregulated (*n *= 23) following ligand treatment and those that were downregulated (*n *= 60). Analysis results revealed that these two groups of genes are involved in distinctly different biological processes (Table 1). The upregulated gene set is significantly enriched for genes that function in lipid and cholesterol transport and metabolism, consistent with known functions of LXRs and their ligands. The downregulated gene set, on the other hand, is overwhelmingly enriched for genes that are involved in cell cycle regulation, DNA replication, and other related biological processes associated with cell proliferation. These findings indicate that ligand-activated LXRs may block cell proliferation by downregulating genes that are involved in processes critical for cancer cell division, and the metabolic functions and the anti-proliferative effects of LXRs are likely regulated via distinctive transcriptional regulatory mechanisms.

**Table 1:** Top fifteen over-represented gene ontology (GO) categories of liver × receptor ligand up- and downregulated genes

GO biological processes	*P*-value
**Upregulated**	
Response to high density lipoprotein stimulus	1.37E-06
Intracellular cholesterol transport	4.57E-06
Cholesterol homeostasis	6.55E-06
Negative regulation of cholesterol storage	6.85E-06
Phospholipid homeostasis	9.59E-06
Positive regulation of cholesterol efflux	1.64E-05
Response to endoplasmic reticulum stress	3.55E-05
Negative regulation of macrophage derived foam cell differentiation	4.14E-05
Phospholipid efflux	4.14E-05
Glutamine metabolic process	7.77E-05
Reverse cholesterol transport	7.77E-05
Positive regulation of protein catabolic process	8.63E-05
Response to nutrient	1.08E-04
Cholesterol efflux	1.47E-04
Endoplasmic reticulum unfolded protein response	1.59E-04
**Downregulated**	
DNA replication	1.66E-48
Cell cycle	5.03E-21
DNA repair	3.75E-18
Nucleotide-excision repair, DNA gap filling	3.52E-13
DNA-dependent DNA replication initiation	2.84E-12
Cell division	1.09E-11
Mitosis	1.41E-08
Response to DNA damage stimulus	2.93E-08
Phosphoinositide-mediated signaling	1.44E-06
DNA unwinding involved in replication	3.23E-06
Modification-dependent protein catabolic process	1.05E-05
Protein K6-linked ubiquitination	1.99E-05
Nucleobase, nucleoside, nucleotide and nucleic acid metabolic process	2.12E-05
DNA strand elongation involved in DNA replication	4.95E-05
Response to organophosphorus	4.95E-05

### Potential role of E2F2 in anti-proliferative effects of LXR ligands

The apparently coordinated downregulation of genes associated with cell growth and proliferation by LXR ligand treatment suggests the targeting of key TFs by activated LXRs. To determine whether downregulated responsive genes shared common *cis*-regulatory sequence motifs, we utilized the Pathway Studio software package to examine their promoter regions (2 kb up- or downstream of the transcriptional start sites) for known TF binding-site motifs. The only sequence motif that showed significant over-representation in the promoter regions of this gene set belongs to the E2F family of TFs.

**Figure 2:**
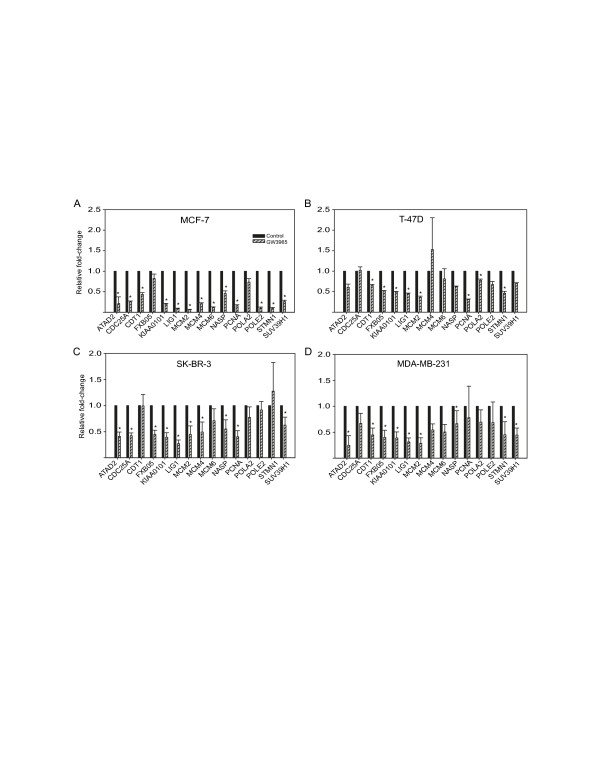
**Expression of predicted E2F target genes responsive to liver × receptor (LXR) ligand treatment was validated by quantitative polymerase chain reaction (qPCR)**. (**A**-D) Expression of fifteen ligand-responsive E2F target genes in control and LXR ligand-treated cells were validated by qPCR in all four cell lines. Significance was determined by Student's *t*-test (*P *< 0.05), and the error bars represent standard error of means from three replicate experiments.

**Figure 3:**
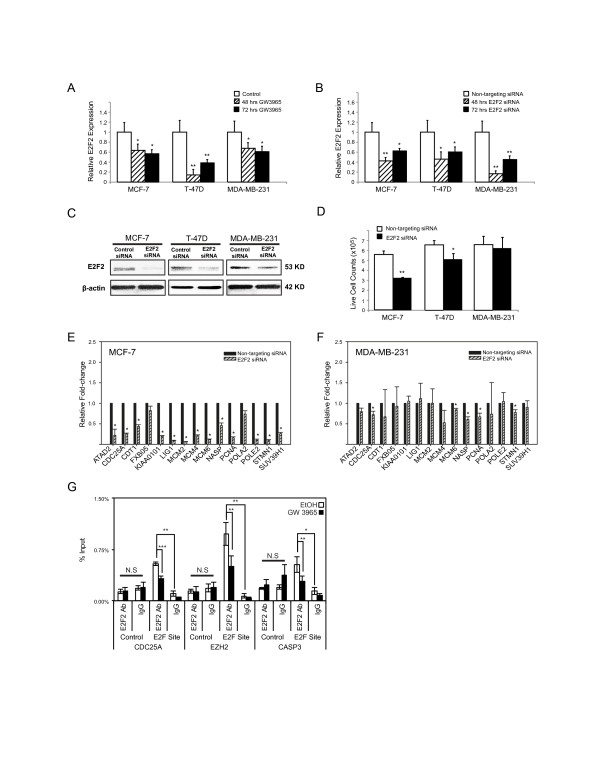
**Knockdown of E2F2 decreased cell growth in estrogen receptor-positive MCF-7 and T-47D cells but not estrogen receptor-negative MDA-MB-231 cells**. (**A**) E2F2 mRNA levels decreased in MCF-7, T-47D and MDA-MB-231 cells following GW3965 treatment. (**B**) E2F2 mRNA levels decreased following transfection with targeting siRNA. (**C**) Protein expression of E2F2 following siRNA transfection at 72 hours post-transfection. (**D**) Trypan blue staining shows a decrease in MCF-7 and T-47D, but not MDA-MB-231 cell numbers following transfection with siE2F2 compared to control siRNA. (**E**) Relative mRNA expression of predicted E2F target genes after E2F2 siRNA transfection in MCF-7 cells. (**F**) Relative mRNA expression of predicted E2F target genes after E2F2 siRNA transfection in MDA-MB-231 cells. (**G**) E2F2 binding to promoter regions of CDC25A, EZH2, and CASP3 in MCF-7 cells was analyzed by chromatin immunoprecipitation assays. Disruption of E2F2 binding was observed following 24 hours of liver × receptor ligand treatment (filled bars) compared to vehicle-treated controls (open bars). No enrichment of binding or disruption following ligand treatment was observed in control immunoprecipitations using pre-immune immunoglobulin or in negative control regions in the exons of target genes. Significance was determined by Student's *t*-test (*P < 0.05, **P < 0.025, ***P < 0.01) and the error bars represent standard error of means from three replicate experiments.

E2F family members are known to regulate the expression of genes that function in cell cycle regulation and DNA replication [32,33]. Strikingly, 15 out of the 60 (25%) downregulated genes identified in our microarray study contain E2F binding-site motifs in their promoter regions. We performed qPCR analysis of their expression to confirm their downregulation by LXR ligand treatment, and found statistically significant (*P *< 0.05, Student's *t*-test) downregulation in the majority of the predicted E2F target genes in all cell lines (Figure 2). To identify the specific E2F family member that might be regulated by LXR ligands and is involved in mediating their anti-proliferative effects, we examined the ligand-responsive microarray data and found E2F2 among the 60 commonly downregulated genes. Time-course analysis of E2F2 transcript levels by qPCR showed decreases of approximately 30 to 40% compared to vehicle-treated controls for up to 72 hours following ligand treatment in MCF-7 cells (Figure 3A). T-47D cells showed the most robust decrease in E2F2 transcript following GW3965 treatment, with over 80% decrease after 48 hours. ER- cell line MDA-MB-231 showed a moderate decrease in E2F2 transcript levels at 48 and 72 hours.

The observed corresponding decreases of E2F2 and E2F target gene transcript levels suggest a possible mechanism by which ligand-activated LXRs can block cell proliferation. To determine the role of E2F2 in breast cancer cell proliferation, we carried out RNA interference experiments in MCF-7 and T-47D (ER+) and MDA-MB-231 (ER-) cells and measured the effects of E2F2 knockdown, mimicking the effects of LXR ligand treatment, on cell numbers over a 72-hour period. Transfection of MCF-7 and T-47D cells with E2F2 targeting siRNAs resulted in approximately 40 to 60% decrease in E2F2 transcript levels for the ER+ cell lines and 60 to 80% decrease in the ER- MDA-MB-231 cells (Figure 3B) for the duration of the study compared to the control siRNA transfected cells. Even though knockdown of E2F2 transcripts was most efficient in MDA-MB-231 cells, protein levels of E2F2 at 48 hours were not significantly changed for the ER- cell line (Figure 3C). However, in the two ER+ cell lines MCF-7 and T-47D, protein levels on E2F2 were significantly decreased after 48 hours. Effects of E2F2 knockdown on cell counts were significantly different from control cells at 72 hours for ER+ cells lines only (Figure 3D). These findings demonstrate that knockdown of E2F2 expression was sufficient to disrupt MCF-7 and T-47D cell proliferation and provide evidence for its role in the mechanisms of action of LXR ligands in ER+ breast cancer cells.

In contrast, knockdown of E2F2 expression (Figure 3B) did not affect MDA-MB-231 cell proliferation (Figure 3D), and this is likely due to the stability of E2F2 proteins in spite of knockdown of E2F2 transcripts. Correspondingly, when we examined mRNA levels of E2F target genes in MCF-7 and MDA-MB-231 cells, 13 out of 15 genes were downregulated in MCF-7 (Figure 3E), but only 5 out of 15 E2F target genes were downregulated in MDA-MB-231 cells (Figure 3F). Of the E2F family members, only E2F2 is downregulated in MDA-MB-231 cells following LXR ligand treatment, whereas in MCF-7 and T-47D, E2F7 is also downregulated (Additional file 4). Members of the E2F TF family are known to act in conjunction or in opposition to each other. In MDA-MB-231, E2F2 is downregulated following GW3965 treatment, but E2F5 is upregulated. In the other two ER+ cell lines, E2F2 and E2F7 are both downregulated following treatment. These results likely reflect the genetic and mechanistic heterogeneity in these cell lines as well as molecular and clinical differences between ER+ and ER- breast cancer cells.

To test the possible direct regulation of predicted E2F target genes by E2F2, we preformed ChIP analysis in MCF-7 cells and observed strong binding of E2F2 to the predicted binding site in the promoter region of the CDC25A gene, and the binding was comparable to known E2F2 binding sites adjacent to CASP3 and EZH2 (Figure 3G). Moreover, treatment with the LXR agonist GW3965 significantly reduced E2F2 binding to all three target gene sites compared to vehicle-treated controls. No enrichment of binding sites and reduction in binding were observed in control experiments using pre-immune immunoglobulin G (IgG) and in exonic regions of target genes. These results suggest that downregulation of E2F2 expression and activity in ER+ breast cancer cells is a potential mechanism by which cell cycle progression and cell proliferation are disrupted following treatment with LXR agonist GW3965 [20].

### Expression profiles of the LXR ligand-downregulated target genes are associated with disease outcome in breast cancer patients

Microarray analysis of LXR ligand-responsive transcriptomic changes in breast cancer cells uncovered 83 genes with transcript levels that were altered in all four breast cancer cell lines selected for this study (Figure 1). Their conserved responses in cell lines representing diverse genetic backgrounds and pathologies suggest that LXR ligands may affect gene networks commonly utilized in breast cancers. To determine the relevance of these LXR ligand-responsive genes in breast cancer, we examined their expression in breast tumors and correlation with disease outcomes in a previously described cohort of 258 breast cancer patients from Uppsala, Sweden [25]. Because the upregulated (*n *= 23) and downregulated (*n *= 60) ligand-responsive genes appear to have distinct functions (Table 1), their expression in tumor samples was analyzed separately.

**Figure 4:**
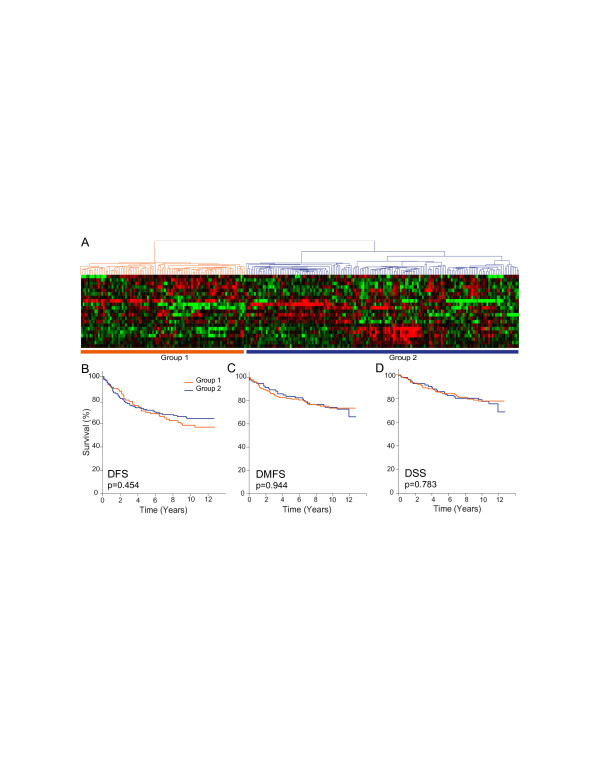
**Expression profiles of commonly upregulated liver × receptor ligand-responsive genes clustered breast cancer patients into two groups with similar disease outcomes**. (**A**) Hierarchical clustering of 258 breast cancer patients by their expression profiles of 23 commonly upregulated genes defined two major patient groups. Kaplan-Meier analysis was carried out on two groups of patients defined by hierarchical clustering for (**B**) disease-free survival (DFS), (**C**) distant-metastasis-free survival (DMFS), and (**D**) disease-specific survival (DSS). Statistical significance of the differences between the two survival plots was determined by the log-rank test and indicated by the resulting *P*-value.

**Figure 5:**
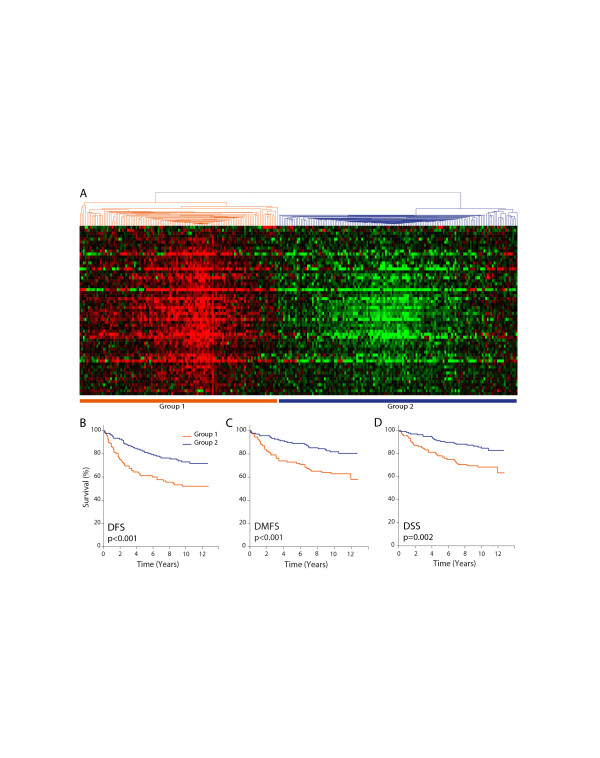
**Expression profiles of commonly downregulated liver × receptor ligand-responsive genes clustered breast cancer patients into two groups with good and poor disease outcomes**. (**A**) Hierarchical clustering of 258 breast cancer patients by their expression profiles of 60 commonly downregulated genes defined two major patient groups. Kaplan-Meier analysis was carried out on two groups of patients defined by hierarchical clustering for (**B**) disease-free survival (DFS), (**C**) distant-metastasis-free survival (DMFS), and (**D**) disease-specific survival (DSS). Statistical significance of the differences between the two survival plots was determined by the log-rank test and indicated by the resulting *P*-value.

Hierarchical clustering of patients based on the expression profiles of the 23 LXR ligand-upregulated genes grouped them into two major clusters (Figure 4A). Kaplan-Meier analysis of patient disease-free survival (DFS), distant metastases-free survival (DMFS), and disease-specific survival (DSS) showed no differences in disease outcomes between the two groups (Figure 4B-D). Clustering of patients with downregulated responsive genes (Figure 5A), however, resulted in two groups with statistically significant differences in DFS, DMFS, and DSS (Figure 5B-D). Specifically, the group of patients whose tumors expressed lower levels of the downregulated genes, similar to the effects of LXR ligand treatment, had better outcomes compared to the group with higher expression levels of these genes. Taken together, these results suggest that LXR ligands target genes that are involved in key processes in breast cancer biology and that may play important roles in determining disease progression, response to therapy, and ultimately, patient survival.

## Discussion

In this study, we performed microarray experiments to determine changes in gene expression associated with the anti-proliferative effects of LXR ligands in breast cancer cells. Our goals were to carry out more comprehensive characterization of the functions of LXRs and their ligands in cancer cells, and to identify their mechanisms of action in order to further evaluate their utility as a potential target mechanism and therapeutic agent, respectively, in the treatment of breast cancer. The four cell lines selected for this study are well-characterized and represent molecularly and clinically diverse cellular models of breast cancer, and the proliferation of all four was blocked by LXR ligand treatment [20]. In addition to their genotypic differences, including both heritable variations and somatic mutations, these cell lines differ in their initial tumor histology, ER status, p53 status, HER2/neu status, responses to targeted therapies and chemotherapeutic compounds, and their tumorigenic and metastatic potential. It is not surprising then that they exhibited vastly different molecular responses to LXR ligand treatment in terms of the number and identity of genes that were responsive to the ligand (Figure 1), as well as the magnitude of change for those genes that were commonly responsive between cell lines (Additional file 3). These differences likely reflect genetic and epigenetic variations between the cell lines, which affect the chromatin structure, the repertoire and functions of co-regulatory proteins and TFs, including LXR subtypes and expression levels, and post-transcriptional regulatory mechanisms, such as those involving microRNA.

In spite of their significant differences in transcriptional responses, however, the four breast cancer cell lines in this study do share a core set of 83 LXR ligand-responsive genes (Figure 1), including those that might be responsible for the anti-proliferative effects of the ligand observed in the cell lines [20]. These genes can be divided into two groups (Table 1) based on their up- or downregulation by ligand treatment, and each group appears to carry out distinct LXR functions. The list of 23 upregulated genes includes known LXR target genes that function in lipid and cholesterol transport and metabolism, whereas the 60 downregulated genes include many that function in cell cycle regulation and DNA replication. This clear delineation of responses and functions suggests the involvement of discrete mechanisms that can potentially be exploited in evaluating existing ligands and in developing novel ligands specifically for cancer treatment. For example, an ideal LXR ligand for targeting cancer cells would have minimal effects on the 23 upregulated responsive genes and their metabolic functions, thus bypassing the undesired increases in plasma and liver triglyceride levels seen in mouse atherosclerosis models following treatments with some LXR agonists [34,35], and elicit robust responses in the 60 downregulated responsive genes associated with cancer cell growth and proliferation.

To further determine the mechanisms of action of LXR ligands on cancer cell proliferation, we analyzed the *cis*-regulatory sequences of the 83 universal LXR ligand-responsive genes identified in our microarray study for the presence of TF binding-site motifs, which may provide clues to factors that may be involved in the observed changes in gene expression. Only the E2F binding-site sequence motif is significantly enriched and only in the promoter regions of the set of 60 downregulated responsive genes. Correspondingly, one of the most downregulated responsive genes was E2F2 (approximately 2.5- to 8.0-fold decrease following ligand treatment). Knockdown of E2F2 expression by RNA interference disrupted ER+ breast cancer cell proliferation (Figure 3D), indicating that downregulation of E2F2 expression by ligand activation of LXRs is a potential mechanism of action for LXR ligands and their anti-proliferative effects in ER+ breast cancer cells. E2F2 RNA interference showed significant decrease of some of the predicted E2F target genes in both ER+ (Figure 3E) and ER- cell lines (Figure 3F). Further ChIP anaylsis showed that indeed in ER+ MCF-7 cells, E2F2 binds to the response element of CDC25A (Figure 3G), and this binding is disrupted following treatment with the LXR ligand GW3965. CDC25A is a cell-division cycle gene whose phosphatase action is required for cell progression from the G1 phase of the cell cycle to the S phase. It has previously been shown that decreases in proliferation of both MCF-7 and Vcr-R, another human breast cancer cell line, after treatment of natural tetrasulfides, are due to inhibition of CDC25 [36]. Furthermore, overexpression of CDC25A in small breast carcinomas is associated with poor survival in patients [37].

It is not clear, however, whether E2F2 is directly targeted by LXRs or through an indirect mechanism. When we examined the genomic regions proximal to the E2F2 gene for LXR response-element sequence motifs, we did not detect any candidate LXR binding-site. This can be due to the sensitivity of the position weight matrix model we used, or the response element for E2F2 regulation might be located in a distal enhancer region not included in our analysis. Activated LXRs may also indirectly regulate E2F2 expression by tethering to another TF or by regulating the expression of another factor, which in turn affects the expression of E2F2. Alternatively, the expression of E2F2 and the other downregulated responsive genes may merely reflect the disruption of cell cycle progression and the observed accumulation of G1/G_0 _cells compared to the cycling vehicle-treated control cells as seen in a previous study. This, however, is unlikely because the changes in gene expression assayed in this study were carried out 48 hours after ligand treatment, whereas the differences in cell cycle progression were observed at 72 hours [20]. The effects of LXR ligands on cancer cells may also involve non-genomic mechanisms, which can regulate cell proliferation and gene expression via post-translational modifications and signal transduction pathways. These hypotheses regarding the mechanisms of action of LXR ligands in cancer cells and the role of E2F2 and other E2F family members in mediating their anti-proliferative effects await further testing and investigation.

The ultimate goals of this study are to understand and exploit the potential impact of LXR ligands on breast cancer progression and patient survival. This study has defined a set of genes whose expression levels were altered in response to LXR ligand treatment in four cell line models of breast cancer. Expression profiles of these genes in breast tumors from a clinically diverse cohort of 258 breast cancer patients were examined to assess their *in vivo *relevance [25]. Hierarchical clustering of patients with the expression profiles of the 60 downregulated responsive genes separated them into two groups with statistically significant differences in disease outcomes (Figure 5). Patients whose tumors expressed lower levels of these 60 genes experienced longer survival times than patients in the higher expression cluster. In addition to disease outcomes, patients in these groups also differed in important clinical parameters, such as ER status, PR status, lymph node status, and tumor grade (Table 2), consistent with the known association of these parameters with disease outcomes. This strong association of the 60-gene signature with patient survival and clinical parameters indicates that LXR ligand treatment elicited transcriptional responses in breast cancer cells similar to expression profiles observed in tumors from patients with significantly better outcomes. Whether LXR ligand treatment of patients will alter the transcriptional programming in tumors and, ultimately, tumor growth and disease outcome, remain to be determined clinically.

**Table 2:** Association of patient clustering by downregulated responsive genes with clinical parameters

Status	Group 1 (*n *= 117)	Group 2 (*n *= 141)	Fisher's exact *P*-value
ER+, n	88	131	0.00001128
PR+, n	71	121	0.00000411
LN+, n	49	35	0.00183468
Tumor grade 3, n	50	5	0.0

ER, estrogen receptor; PR, progesterone receptor; LN, lymph node; n, number of patients.

## Conclusions

LXR ligands target gene networks critical for cell growth and proliferation and disease progression, including those regulated by E2F transcription factors, and warrant further study and consideration as potential agents in the prevention and treatment of breast cancers.

## Abbreviations

ChIP: chromatin immunoprecipitation; ct: cycle threshold; DFS: disease-free survival; DMEM: Dulbecco's modified Eagle's medium; DMFS: distant metastases-free survival; DMSO: dimethyl sulfoxide; DSS: disease-specific survival; ER: estrogen receptor; FBS: fetal bovine serum; GO: gene ontology; HER: human epidermal growth factor; IgG: immunoglobulin G; LXR: liver × receptor; NR: nuclear receptor; PBS: phosphate-buffered saline; PCR: polymerase chain reaction; PR: progesterone receptor; PVDF: polyvinylidene fluoride; qPCR: quantitative polymerase chain reaction; RPMI: Roswell Park Memorial Institute; RXR: retinoic acid receptor; TBST: Tris-buffered saline and Tween 20; TF: transcription factor.

## Competing interests

The authors declare that they have no competing interests.

## Authors' contributions

TNV performed the microarray data acquisition, data analysis and mining, experimental validation and hypothesis testing, and contributed to the drafting of the manuscript. LLV obtained the samples and performed western analysis. KL designed and carried out functional assays. PJ was responsible for data mining using pathway analysis tools. JZL participated in sample preparation and microarray data acquisition and analysis. NRC performed the ChIP experiments. LPC performed qPCR validation of microarray data. SA contributed to the validation of microarray data. CW participated in the conception and design of the study and drafting of the manuscript. JÅG was involved in the conception of the study and the drafting of the manuscript. KRS conceived the study, coordinated sample acquisition, and helped draft the manuscript. CYL conceived the study, provided overall coordination of the study, and drafted the manuscript. All authors read and approved the final manuscript.

## Supplementary Material

Additional file 1 **Primer sequences used for qPCR and ChIP analyses**. Forward and reverse primer sequences of genes used in quantitative (q)PCR and chromatin immunoprecipitation (ChIP) confirmations. Click here for file

Additional file 2 **Relative liver × receptor (LXR)a and LXRb expression**. (**A**) Expression of LXRa in four different breast cancer cell lines without (control) and with (GW-treated) LXR agonist. (**B**) Expression of LXRb in the same four cell lines. Click here for file

Additional file 3 **Common LXR ligand-responsive genes in all four cell lines**. Fold-changes and *P*-values for common liver × receptor (LXR) ligand-responsive genes in MCF-7, T47D, SK-BR-3, and MDA-MB-231 cell lines. Click here for file

Additional file 4 **Expression of E2F family members in breast cancer cell lines following liver × receptor (LXR) ligand treatment**. Directional expression of different E2F family members from the microarray data following LXR ligand treatment in MCF-7, T47D, SK-BR-3, and MDA-MB-231 cell lines. Click here for file
