# Supplementary material for: Liver × receptor ligands disrupt breast cancer cell proliferation through an E2F-mediated mechanism
Source: Breast Cancer Res. 2013 Jun 20;15(3):R51. doi: 10.1186/bcr3443 (PMC4053202; doi:10.1186/bcr3443)
Supplement: Additional file 2 — Relative liver × receptor (LXR)a and LXRb expression. (A) Expression of LXRa in four different breast cancer cell lines without (control) and with (GW-treated) LXR agonist. (B) Expression of LXRb in the same four cell lines. [file bcr3443-S2.PDF]

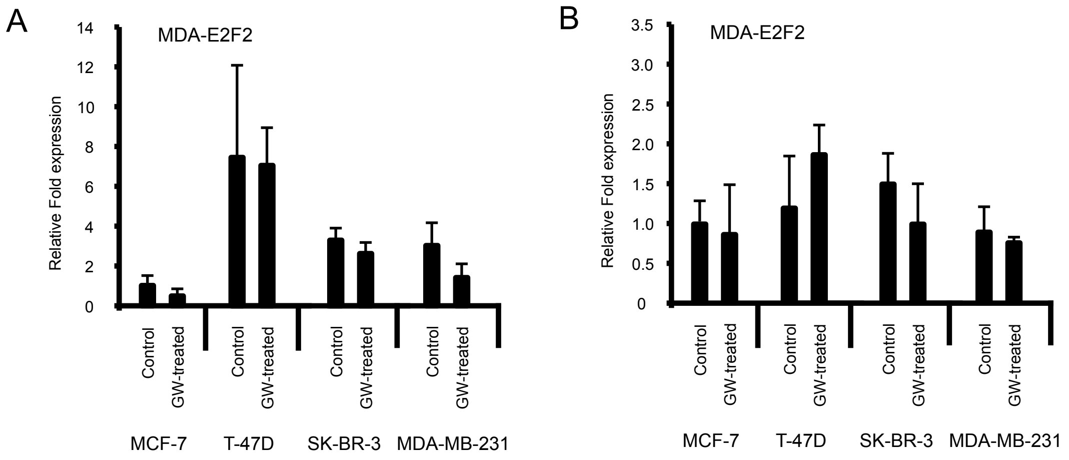

Addition file 2. Relative LXRa and LXRb expression. A) Expression of LXRa in four different breast cancer cell lines without (control) and with (GW-treated) LXR agonist. B) Expression of LXRb in the same four cel lines.
