# Supplementary material for: Liver × receptor ligands disrupt breast cancer cell proliferation through an E2F-mediated mechanism
Source: Breast Cancer Res. 2013 Jun 20;15(3):R51. doi: 10.1186/bcr3443 (PMC4053202; doi:10.1186/bcr3443)
Supplement: Additional file 4 — Expression of E2F family members in breast cancer cell lines following liver × receptor (LXR) ligand treatment. Directional expression of different E2F family members from the microarray data following LXR ligand treatment in MCF-7, T47D, SK-BR-3, and MDA-MB-231 cell lines. [file bcr3443-S4.PDF]

	MCF-7	T-47D	SK-BR-3	MDA-MB-231
E2F1				
E2F2	↓	↓	↓	↓
E2F3a			↓	
E2F3b			↓	
E2F4				
E2F5	↑			↑
E2F6				
E2F7	↓	↓	↓	
E2F8				

Addition file 4. Expression of E2F family members in breast cancer cell lines following LXR ligand treatment.
